# Human APOBEC3G-mediated hypermutation is associated with antiretroviral therapy failure in HIV-1 subtype C-infected individuals

**DOI:** 10.7448/IAS.16.1.18472

**Published:** 2013-02-25

**Authors:** Ujjwal Neogi, Anita Shet, Pravat Nalini Sahoo, Irene Bontell, Maria L Ekstrand, Akhil C Banerjea, Anders Sonnerborg

**Affiliations:** 1 Unit of Infectious Diseases, Department of Medicine, Karolinska Institutet, Stockholm, Sweden; 2 Clinical Virology, Department of Microbiology, St. John's Medical College, Bangalore, India; 3 Department of Pediatrics, St. John's Medical College Hospital, Bangalore, India; 4 Department of Medicine, Center for AIDS Prevention Studies, University of California, San Francisco, CA; 5 Virology, National Institute of Immunology, New Delhi, India; 6 Division of Clinical Virology, Department of Laboratory Medicine, Karolinska University Hospital, Stockholm, Sweden

**Keywords:** APOBEC3G/F, hypermutation, drug resistance, antiretroviral therapy, India

## Abstract

**Introduction:**

Human APOBEC3G/F (hA3G/F) restricts retroviral replication through G-to-A hypermutations, which can generate drug-resistant progenies *in vitro*. The clinical relevance is still inconclusive. To bridge this gap, we aim to study the role of these hypermutations in evolution of drug resistance; we characterised hA3G/F-mediated hypermutations in the RT region of the *pol* gene of patients with or without antiretroviral therapy (ART).

**Methods:**

In 88 HIV-1-positive individuals, drug resistance genotyping was carried out in plasma virus and provirus by population sequencing. Hypermutations were determined by three different approaches using Hypermut 2.0 software, cluster analysis and APOBEC3G-mediated defectives indices. Clinical and demographic characteristics of these individuals were studied in relation to these hypermutations.

**Results:**

hA3G/F-mediated hypermutated sequences in proviral DNA, but not in plasma virus, were identified in 11.4% (10/88) subjects. Proviral hypermutations were observed more frequently in patients with ART failure than in ART-naïve individuals (*p*=0.03). In therapy failure patients, proviral hypermutation were associated with greater intra-compartmental genetic diversity (*p*<0.001). In therapy-naïve individuals, hypermutated proviral DNA with M184I and M230I mutations due to the editing of hA3G, had stop codons in the open reading frames and the same mutations were absent in the plasma virus. Only a limited concordance was found between the drug resistance mutations in plasma RNA and proviral DNA.

**Conclusions:**

hA3G lethal hypermutation was significantly associated with ART failure in Indian HIV-1 subtype C patients. It is unlikely that viral variants, which exhibit hypermutated sequences and M184I and/or M230I, will mature and expand *in vivo*.

## Introduction

The human apolipoprotein B mRNA-editing enzyme catalytic polypeptide-like 3G (APOBEC3G or hA3G) belongs to a family of at least 10 other proteins including hA3D, hA3F, hA3H which acts as a potent host restriction factor of retroviral replication through cytidine deaminase activity [1, 2]. HIV-1 accessory protein *vif* interacts with hA3G and protects the virus from its anti-viral activity [3–5]. In the presence of defective *vif*, hA3G/F/D/H induces extensive dC-to-dU mutations in the minus strand of the single stranded DNA [6], consequently, dG-to-dA mutations in the plus strand of the cDNA [3, 7]. Generally, hA3G restricts viral replication through lethal hypermutations by introducing stop codons into the open reading frame (ORFs) of the retroviral gene mainly in the tryptophan residue (T**GG**-to-T**GA**/T**AA**/T**AG**). However, sub-lethal hypermutations have been suggested to contribute to the HIV-1 genetic diversity [8] and the low level of G-to-A mutation allows for greater genetic variations affecting HIV-1 evolution [9].

Cytidine deamination in the proviral sequences can generate drug-resistant progenies *in vitro* [10], though the *in vivo* consequences of hA3G/F are not well understood. Computer prediction of *pol* sequences has identified potential target sites for hA3G/F, but the role of hA3G in HIV-1 drug resistance *in vivo* is unknown and considered to be low [9]. Also, most of the previous *in vivo* studies used HIV-1 genes *gag*, *env*, *vpu*, and *vif* to identify hA3G induced hypermutations in proviral sequences [11–13]. Only a few studies have analysed the HIV-1 *pol* gene, which is a major target in antiretroviral therapy (ART) [14–16].

To bridge the gap between the *in vitro* observations and the limited knowledge about the *in vivo* consequences, we aimed to characterise the nature of hA3G/F-mediated hypermutations in the RT region of the *pol* gene in a population of Indian HIV-1-positive patients, and its correlation with clinical and demographic parameters as well as with drug resistance.

## Methods

### Patient populations

Blood samples were collected in EDTA tubes (BD, USA) from 102 HIV-1-positive individuals who were participants of on-going studies in southern India between November 2009 and October 2011. Among the 102 patients, both RNA virus and provirus were amplified in 86.2% (88/102), only RNA virus in 5.9% (6/102) and only provirus in 7.8% (8/102). Only paired sequences from both RNA virus and provirus (*n*=88) were included in the study. Among these patients, 56 were ART naive and 32 were ART experienced at inclusion in the study (Table 1). In the treatment-experienced patients, the CD4 + T-cell values were <250 cells/µl when ART was initiated, according to the Indian guidelines. The experienced patients were on first-line therapy with two nucleoside RT inhibitors (zidovudine or stavudine with lamivudine) and one non-nucleoside RT inhibitor (nevirapine or efavirenz). Routine CD4 + T-cells were measured with FACSCalibur system (BD, USA). Viral load was measured every sixth month by Abbott m2000rt system (Abbott Molecular Diagnostics, US). In the treated patients, peripheral blood samples for this study were obtained at the first virological rebound (median duration 29 months; IQR: 12–44 months).

**Table 1:** Clinical and demographic data of all study subjects (*n*=88) with hypermutated (*n*=10) or non hypermutated (*n*=78) proviral sequences

Parameter	All patients (*n*=88)	Hypermutated (*n*=10)	Non-hypermutated (*n*=78)	p*
Age				
Years median (IQR)	36 (30–41)	33 (29–37)	37 (30–42)	0.11**
Sex male, No (%)	56 (63.6%)	7 (70%)	49 (62.8%)	0.74***
Route of transmission				
Heterosexual	69 (78.4%)	7 (70%)	62 (79.5%)	0.66***
Perinatal	15 (17%)	2 (20%)	13 (16.7%)	
Other/unknown	4 (4.5%)	1 (10%)	3 (3.8%)	
CD4 T-cells/mm^3^				
Median (IQR)	208 (117–363)	262 (154–485)	199 (98–356)	0.14**
HIV RNA log_10_ copies/ml				
Mean (SD)	5.03 (0.86)	4.79 (0.75)	5.05 (0.88)	0.25**
Treatment, No (%)				
Yes	32 (36.3%)	7 (70%)	25 (32.1%)	
No	56 (63.6%)	3 (30%)	53 (67.9%)	0.03***
HIV-1 subtype	C (98.9%) A1C (1.1%)	C (100%)	C (98.7%) A1C (1.3%)	–

CD4 T-cells and HIV RNA analysed at therapy failure in treatment experienced patients. The CD4 T-cells were <250 cells/µl, when ART was initiated; all treated patients were given first-line therapy (zidovudine or stavudine with lamivudine and nevirapine or efavirenz).

* *P* values based on the comparison between patients with hypermutated versus non-hypermutated sequence; the percentage mentioned is the percentage of each subsets

** Mann–Whitney U Test

*** Fisher's exact test. Statistical significance (*p*<0.05) is marked in bold letters.

Statistical significance (*p*<0.05) is marked in bold letters.

### PCR, proviral sequencing and subtyping

Plasma HIV-1 RNA and proviral DNA from whole blood were extracted, amplified and sequenced, using an in-house genotyping assay as described previously [17, 18]. In brief, a partial RT (17–235 aa) region of the *pol* gene was amplified from cDNA and proviral DNA, respectively, by conventional nested PCR. The purified nested PCR products were subjected to bidirectional population sequencing. Sequences were submitted to GeneBank with the following accession numbers: KC307783–KC307958.

HIV-1 subtyping was carried out using maximum likelihood phylogenetic analysis with best-fitted model for the dataset in MEGA 5.0 software [19]. Recombination was identified by the RIP 3.0 program available in Los Alamos Database (www.hiv.lanl.gov).

### Estimation of G-to-A substitutions

To estimate G-to-A substitutions, proviral DNA sequences were aligned against the consensus Indian subtype C sequence [17]. The hA3G/F-mediated GG-to-AG and GA-to-AA scores, respectively, for each sequence were calculated [11]. The consolidated hA3G/F-mediated G-to-A hypermutation score was calculated as: [(Number of GG-to-AG or GA-to-AA substitutions/number of GG or GA in Indian consensus sequence)/(total number of mutations/sequence length)]. G-to-A preferences were calculated as described [11].

### Identification of hypermutated sequences

Hypermut software was used to determine hA3G/F-mediated hypermutated sequences [20]. Further cluster analysis of preference for G-to-A substitutions relative to consolidated hA3G/F score and sequence analysis of 21 hA3G and 20 hA3F target sites in the 17–235 aa of the RT region identified by APOBEC3G-mediated defectives (A3GD) indices [21] were also used. Hypermutations were labelled into a dichotomous variable if identified by one of the methods mentioned. Mutations were designated as lethal if there was stop codon in the ORFs.

### Drug resistance mutations and nucleotide divergence

The World Health Organization (WHO) recommendations for surveillance of drug resistance mutations updated in the 2009 (SDRM_2009) list were used to define the transmitted drug resistance mutations in therapy-naïve patients [22]. Drug resistance mutations in therapy failure patients in the RT region (17–235 amino acids) listed in the December 2011 update from the International AIDS Society were considered [23]. The genetic distance of each of the sequences to the Indian consensus C sequence (intra-population divergence) and the intra-compartmental genetic diversity were calculated in MEGA 5 software [19].

### Statistical analysis

Descriptive statistics were used to describe the characteristics of the patients. The demographic, clinical and viral genetic differences between hypermutated and non-hypermutated groups were evaluated by Mann–Whitney U Test and Fisher's exact test. Spearman rank co-relation was used to find associations between different factors. The statistical analysis was calculated in SPSS software version 16.

### Ethical approval

The study was approved by Institutional Ethical Review Board, St. John's Medical College Hospital, Bangalore, India (IERB Study No. 153/2010). Written informed consent was obtained from all the adult participants and the caregivers of the children prior to recruitment, and a verbal assent was obtained from children older than nine years.

## Results

### Subtyping

Subtype C was identified in 98.9% (87/88) of the patients along with one A1C recombinant strain. Phylogenetic analysis using the sequences from both compartments verified the common origin of the strains in each individual.

### Identification of hA3G-hypermutations

**Figure 1:**
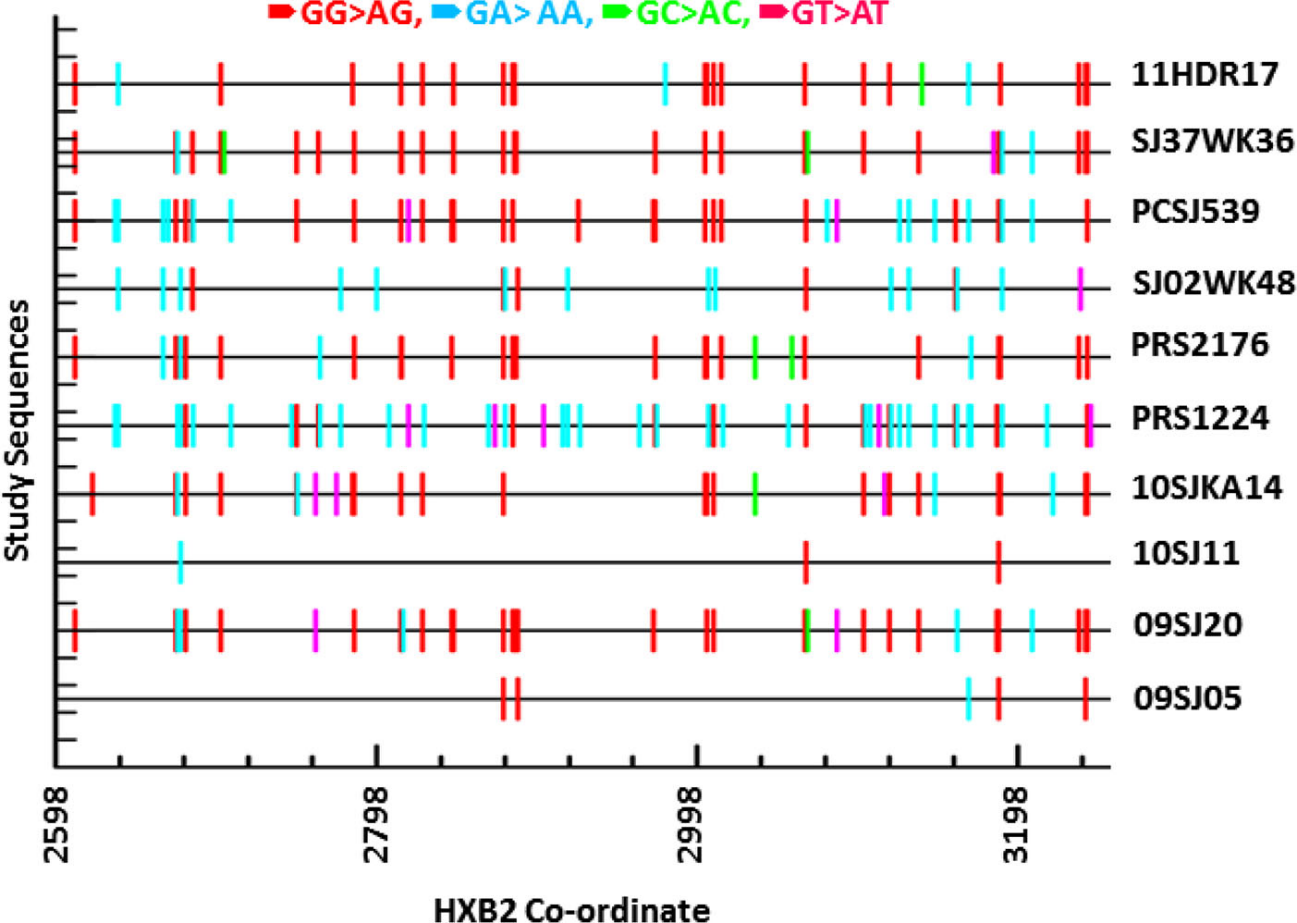
hA3G mediated hypermutations in proviral DNA. **The sequences were run in the HyperMut software with Indian consensus C sequences as a reference sequences. GG to AG mutations were labelled in red, GA to AA in cyan, GC to AC in green, GT to AT in magenta.**

hA3G mediated hypermutated sequences in proviral DNA were identified in 11.4% (10/88) of the patients (Figure 1). However, the event of hypermutation was not detected in the plasma viral RNA. Among the clinical and demographic factors, only treatment failure was associated with hypermutation as compared to naïve patients (Table 1) (*p*=0.03; Fisher's exact test). Patients failing therapy had a 4.96-fold (OR; 95% CI; 1.18, 20.75) higher risk of lethal hypermutations than therapy-naive patients. Among the six therapy-experienced patients whose samples were available before therapy, two had hypermutated proviral sequences at failure but not when they were therapy-naïve.

### Association of G-to-A substitutions to clinical and demographic parameters in therapy-experienced patients

Among therapy-experienced patients, univariate analysis revealed no significant association with respect to patients’ clinical-demographic parameters and the presence of hypermutated or non-hypermutated sequences (Table 2). It is likely that the patients had reached their set point for viral load after failure and it did not differ between those with or without hypermutated sequences. A difference between the hypermutated and non-hypermutated therapy-experienced groups was observed in the hA3G-specific G-to-A score (*p*<0.001) and the intra-compartmental genetic diversity (*p*<0.001), but not in the hA3F-specific G-to-A score (Table 2).

**Table 2:** Clinical, laboratory and viral genetic factors of the patients with ART failure

Parameter	Hypermutated (*n*=7)	Non-hypermutated (*n*=25)	*p*
Age			
Years median (IQR)	35 (23.5–36.5)	38.5 (29–45)	0.18*
Sex			
Male (%)	5 (71.4%)	17 (71%)	0.98**
CD4+ T-cell count/mm^3^			
Median (IQR)	278 (178–638)	268 (172–404)	0.54*
Viral load log_10_ copies/ml			
Mean (SD)	4.36 (4.19–4.96)	4.27 (3.46–4.95)	0.44*
Duration of ART			
Median (IQR)	12 (10–45)	30 (14–38)	0.51*
Consolidated scores mean (SD)			
hA3G G-to-A	7.72 (4.20)	1.80 (0.94)	<0.001*
hA3F G-to-A	2.70 (2.38)	1.36 (0.74)	0.21*
Viral divergence mean (SD)			
Proviral DNA	0.09 (0.04)	0.04 (0.01)	<0.001*
Plasma RNA	0.02 (0.01)	0.03 (0.01)	0.43*
Intra-compartmental genetic diversity			
Median (IQR)	0.05 (0.04–0.07)	0.01 (0.007–0.014)	<0.001*

* Mann–Whitney U Test

** Fisher's exact test.

### Correlation between hA3G mediated hypermutation and viral heterogeneity

When all of the patients were analysed, the consolidated hA3G specific G-to-A score was weakly associated with proviral divergence (Spearman rho = 0.24; *p*=0.02), but not with plasma viral divergence. This correlation was found in the therapy-experienced patients only (Spearman rho = 0.57; *p*=0.001), but not in the therapy-naive individuals.

### Mutations in computer derived hA3G/F motifs

**Figure 2:**
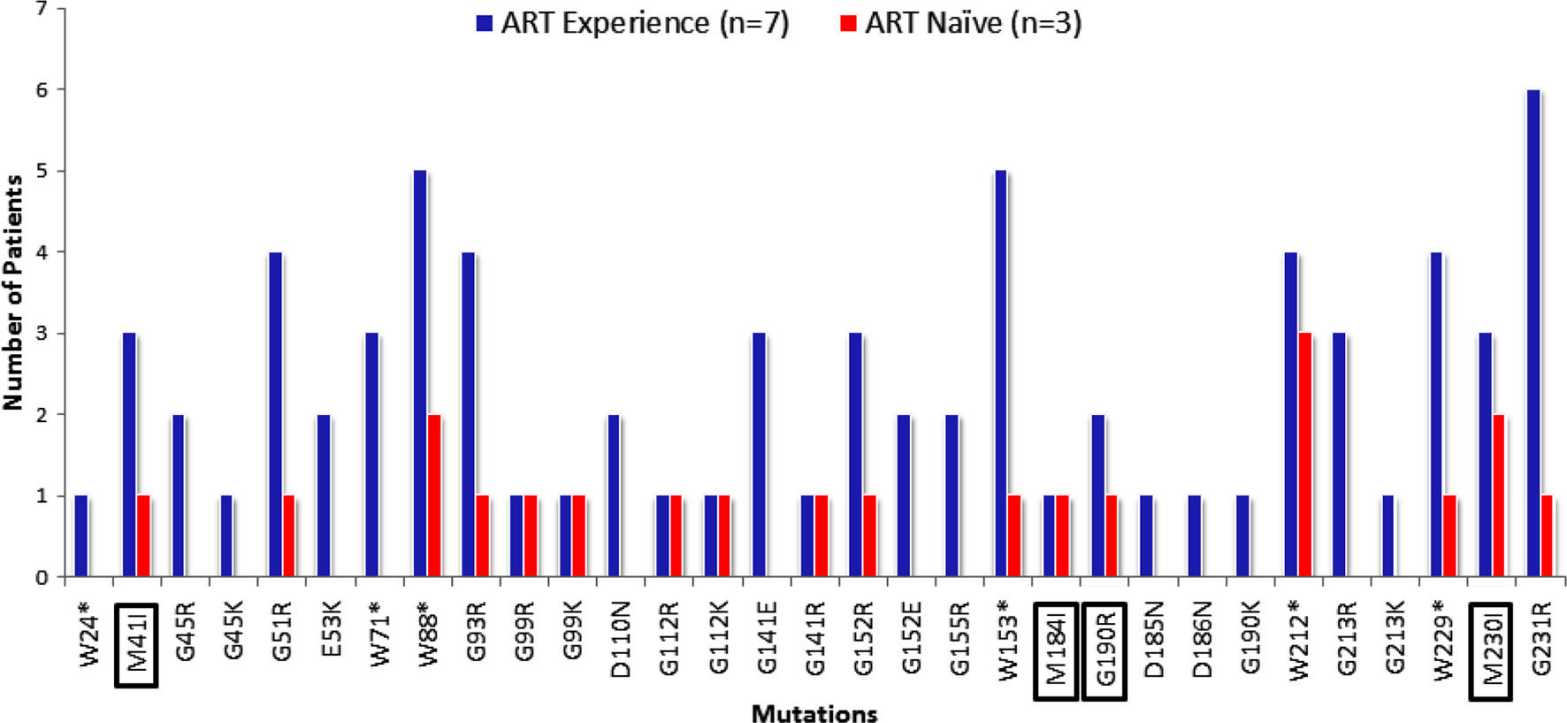
Mutations in hA3G/F target motifs in HIV-1 subtype C patients.** Number of mutations in the hA3G/F target motifs identified by APOBEC3G-mediated defective (A3GD) indices** [21] were presented. Blue bar represents sequence from therapy-experienced patients and red bar represents therapy naive patients. “*” indicates stop codon. Clinically important drug resistance mutations positions are marked with a box.

Among the hypermutated proviral DNA sequences, substitutions were observed in 31 aa residues, mainly in glycine and tryptophan (Figure 2). None of these mutations have been previously reported in HIV-1 subtype C, using Stanford Database HIVseq program. The three hypermutated proviral sequences from the therapy naive patients who had M184I and M230I drug resistance mutations and mutation in drug resistance position (M41I) also had stop codons in the RT ORF particularly at the tryptophan residue, which is the target site for hA3G.

### Drug resistance mutations

Among the 56 treatment naïve patients, transmitted drug resistance mutations (DRM) were observed in both compartments of one patient giving the prevalence of transmitted drug resistance as 1.8% (1/56). One patient had D67DN and K70KE mutations in the proviral sequence. In one patient with hypermutated sequence, M184I was observed in the proviral sequence but not in plasma. Therefore, a high level of concordance in DR Genotyping (94.6%, 53/56) was observed in the therapy-naïve individuals.

DRM were found in 84.4% (27/32) of the patients failing ART (Table 3). Exactly the same DRM in the RNA and DNA sequences were observed in 43.8% (14/32) of patients. As expected, among the NRTI and NNRTI mutations, M184I/V (71.9%), T215Y/F (34.4%), K103N (34.3%), and Y181C (28.1%) were the most prevalent in any of the compartments. This discrepancy was not only because of the hA3G restriction. Among the discordant sequence, only three (16.7%; 3/18) had hA3G associated hypermutations in their proviral sequences.

**Table 3:** Drug resistance mutations in plasma RNA and proviral DNA in therapy-experienced patients

Plasma virus			Provirus		
	
	NRTI mutations	NNRTI mutations	NRTI mutations	NNRTI mutations	hA3G
**1**	M41L, D67N, K70R, M184V, T215Y, K219E	Y188L	M41L, D67N, K70R, **V75I**, M184V, T215Y, K219E	Y188L, **M230I**	Yes
**2**	D67N, T215Y, K219E	Y188L	D67N, **K70KR, M184V**, T215Y, K219E	**L100I, Y188L**	
**3**	M184V	Y181C	M184V	Y181C	
**4**	M184V, T215Y, K219Q	K103N, M230L	M184V, T215Y, K219Q	K103N, M230L	
**5**	None	V106A	None	V106A	
**6**	M41L, D67N, K70R, M184V, T215Y	A98G, K101E, G190A	M41L, D67N, K70R, M184V, T215Y	A98G, K101E, G190A	
**7**	D67N, K70R, M184V, K219E	V106M, F227L	D67N, K70R, M184V, K219E	V106M, F227L	
**8**	None	None	None	None	Yes
**9**	K70R, M184V, K219E	K103N, Y181C	**K65KR**, K70R, M184V, K219E	K103N, Y181C	
**10**	**M184V, T215Y**	**K101E, V108I, Y181C**	None	None	
**11**	None	None	None	None	
**12**	M41L, L74V, M184V**, L210W, T215Y**	V108I, Y181C	M41L, L74V, M184V	V108I, **E138K**, Y181C	Yes
**13**	**M184V**, **T215Y**	A98G, **K101E**, G190A	None	A98G, G190A	
**14**	**D67N, K70R**, M184V, **K219E**	**K103N, G190A**	M184V	None	
**15**	M41L, M184V, T215F	A98G, K103N	M41L, M184V, T215F	A98G, K103N	
**16**	**M184V**	**Y181C**	None	None	
**17**	M184V	Y181C	M184V	Y181C	
**18**	M184V	K101E, V106M, G190A	**M41L, D67G**, M184V	K101E, V106M, G190A	
**19**	M184V	K103N	M184V	K103N, **M230I**	Yes
**20**	None	None	None	None	Yes
**21**	None	**K103N**	None	**E138K**	
**22**	None	V106M	None	V106M	
**23**	None	None	**M184I**	**M230I**	Yes
**24**	M184V	K103N	M184V	K103N	
**25**	**M184V**	**K103N**	None	None	
**26**	**M184V**	**Y181C**	None	None	Yes
**27**	**M184V**	**K103N, Y181C**	None	None	
**28**	M41L, M184V, T215Y	K103N, V108I, Y181C, G190A	M41L, **L74V**, M184V, T215Y	K103N, V108I, Y181C, G190A	
**29**	None	None	None	None	
**30**	None	**V106M**	**D67DN**	**G190AG**	
**31**	None	None	None	None	
**32**	M41L, D67N, M184V, L210W	A98G, K101E, **V106IM**, G190A	M41L, D67N, M184V, L210W, **T215Y**	A98G, K101E, G190S	

Discordant mutations are marked bold.

Important differences were found between the plasma RNA and the proviral DNA compartments. M184V and T215Y/F were observed in plasma only in six and three patients, respectively, while M41L and K65R were observed in one patient each in provirus only. Additional NRTI mutations were observed in 25% (8/32) of proviral and plasma viral sequences, respectively. Additional NNRTI mutations were found mainly in plasma (31.2%; 10/32) and to a lesser extent in proviral DNA (12.5%; 4/32). In two proviral sequences only, the E138K mutation was observed.

## Discussion

In the present study, hA3G mediated lethal hypermutations were identified in the clinically important *pol* gene of proviral DNA from a minority (11.4%) of 88 Indian HIV-1 subtype C-infected patients. The hypermutations occurred more frequently in patients failing therapy than in therapy-naïve patients. There was a correlation between their presence and the proviral divergence, which is in line with the view that hA3G contributes to viral evolution.

*In vitro* studies and computer predictions have suggested a role of hA3G in the evolution of HIV drug resistance [9, 10]. Thus, due to a suboptimal anti-APOBEC3G activity of HIV-1 *Vif* mutants, the HIV drug resistance mutations M184I [10, 16] and E138K [16] may be induced *in vitro* without any drug exposure. Also, one study has reported the co-presence of M184I and E138K in 24% of hypermutated sequences of treated patients [16]. In contrast, co-evolution of M184I and E138K was not found in our hypermutated sequences. However, random polymorphisms of E138K/A were observed in the proviral DNA of both therapy-naïve and experienced patients.

In addition to these *in vitro* results, differences have been reported on how often hA3G induced mutations can be found *in vivo*. When analysing HIV clones, hypermutated proviral DNA was detected in resting T-cells of all nine treated patients who had undetectable viremia, suggesting that the mutated viral genomes were able to integrate and persist in these cells [14]. In contrast, a minority (9.4%) out of 127 untreated subtype B infected mainly Caucasians exhibited such mutations at population based sequencing [13]. Furthermore, an even lower prevalence (4.8%) was found in 601 proviral DNA sequences of treated patients derived from a French database [16]. In our study, higher hypermutated proviral DNA was found in patients failing ART, but in naïve patients the incidence was very low. Altogether, these data suggest that hA3G generated mutations are common but are mostly restricted to a minor viral population. However, it also seems possible that, in addition to the different techniques used, either clonal analysis or population based sequencing, the characteristics of patients studied might influence the extent hypermutations are found. Our data indicate that hypermutated proviral DNA accumulates in patients with therapy failure. This is further supported by the increase in intra-compartmental genetic diversity in patients with hypermutated proviral DNA as compared to those without. Also, in a re-analysis of earlier published sequences from India [24], we observed hA3G restriction in only two sequences of therapy-experienced patients. A recent study on five patients with long term successful treatment showed that these five patients harboured more in-frame stop codons in the proviral compartment compared to the therapy-naïve patients [25], thus the effective ART may lead to the accumulation of the defective genomes in the reservoir [25].

No hypermutation in plasma HIV RNA was found. This is in line with the finding that the viruses released into plasma at low levels in nine patients on successful ART were devoid of hypermutated sequences [14]. This data is further strengthened by the observation from the Swedish InfCare HIV cohort in which only three hypermutated RT sequences have been found after analyzing >2000 tested samples (unpublished data). As the hypermutated sequences with DRM (M184I; M230I) had stop codons in the ORFs and the absence of these DRMs in the plasma viral RNA, it is unlikely that such proviral variants will mature and expand *in vivo*. The presence of an increased number of hypermutated sequences in the therapy failure patients might be associated with decreased fitness of the virus due to the DRMs [26] as these strains might be superimposed by the more replication competent viruses compared to the less fit DRMs containing viruses after the treatment was initiated.

In addition to our analysis of hA3G mutations, we compared the sequences with regard to DRM. Transmitted drug resistance was uncommon which is concordant with previous findings [17, 27]. For the failing patients, the DRM were expected [28, 29]. However, we found a low level of concordance between the DRM in plasma and proviral DNA which is in line with the study of Chew et al. where DRM were more common in plasma [4]. In contrast, a study from Honduras showed 88% concordance between both compartments in heavily ART-treated patients [30].

The presence of drug resistance mutations like M184V, T215Y, K103N, and Y181C in plasma virus but not in the provirus in our study indicates that routine HIV RNA monitoring every six months and subsequent plasma viral genotyping at failure identifies the most recent viral populations circulating, although different sources of the two viral populations cannot be excluded. Therefore, in such a setting, testing of proviral DNA could possibly underestimate the true burden of recently developed resistant virus. However, the presence of key mutations in the proviral DNA which was not present in plasma is of importance for the selection of future ART regimen. World Health Organisation recommends the use of dried blood spot for transmitted drug resistance surveillance but not in patients undergoing therapy [31]. High concordance in the DR Genotyping in both the compartment in therapy-naïve patients corroborates the idea. It may solve the logistics challenges in countries where most of the part the cold chain transport is not available. Thus, although routine drug resistance testing is frequently not affordable in resource-poor settings, further studies are recommendable to evaluate which compartment to analyse, especially in patients on therapy.

Our study has some inadvertent caveats. First, the analysis is based on population sequencing and therefore does not detect any hypermutations in the minor quasi-species. Second, the number of therapy failure patients is low compared to the treatment naïve individuals. This is due to a low number of treatment failures in our settings because of high (>95%) adherence. We have thus observed only 2.8% (9/323) of viral treatment failure with a median duration of four years of therapy who had initially suppressed viremia following the initiation of the therapy [32]. Third, we do not know to which extent the mutations were present before initiation of ART since the analysed longitudinal samples from six patients only before and after therapy. However, even in this small sample size, hypermutation developed during therapy failure in two subjects.

## Conclusions

In conclusion, our study showed for the first time that hA3G lethal-hypermutation was associated with the use of treatment in Indian patients who failed ART. Though DRM were present in the hypermutated proviral compartment, all strains had a stop codon in its ORF. Therefore, it is unlikely that such viral variants will mature and expand *in vivo* which is supported by the absence of those mutations in plasma viral populations. However, further studies are required to validate the idea with appropriate *in vitro* cell culture models and with analysis of a large number of patients to gain the mechanistic view of the role of hA3G in the evolution of drug resistance and its clinical consequences. The evidence from this study also suggests the possible use of proviral drug resistance genotyping as an alternate to plasma viral genotyping for surveillance of transmitted drug resistance in resource-limited settings, specifically from the rural and remote part of the country where the logistics challenges remain.
